# miR-34a induces neutrophil apoptosis by regulating Cdc42-WASP-Arp2/3 pathway-mediated F-actin remodeling and ROS production

**DOI:** 10.1080/13510002.2022.2102843

**Published:** 2022-08-06

**Authors:** Meiwan Cao, Baoling Peng, Huan Chen, Min Yang, Peiyu Chen, Liping Ye, Hongli Wang, Lu Ren, Jing Xie, Jingnan Zhu, Xiangye Xu, Wanfu Xu, Lanlan Geng, Sitang Gong

**Affiliations:** a Department of Gastroenterology, Guangzhou Women and Children’s Medical Center, Guangzhou Medical University, Guangzhou, People’s Republic of China; b Center for child health and mental health, Shenzhen Childen’s Hospital, Shenzhen, People’s Republic of China; c Department of Hematology, Guangzhou Women and Children’s Medical Center, Guangzhou Medical University, Guangzhou, People’s Republic of China; d Guangzhou Institute of Pediatrics, Guangzhou Women and Children’s Medical Center, Guangzhou Medical University, Guangzhou, People’s Republic of China

**Keywords:** MiR-34a, myelodysplastic syndromes, neutrophils, apoptosis, ROS, F-actin, remodeling‌, Cdc42-WASP-Arp2/3 pathway

## Abstract

**Background:**

The number of neutrophils is significantly reduced in myelodysplastic syndrome (MDS), but the molecular basis remains unclear. We recently found that miR-34a was significantly increased in MDS neutrophils. Therefore, this study aims to clarify the effects of aberrant miR-34a expression on neutrophil counts.

**Methods:**

miR-34a mimics/inhibitor transfection were performed in neutrophil-like differentiated HL60 (dHL60) cells, and a FACSCalibur flow cytometer was used to measure ROS production and apoptosis. In addition, the Cdc42-WASP-Arp2/3 pathway inhibitor (ML141) and activator (CN02) treated the dHL60 cells, and then ROS production, apoptosis and related proteins expression were detected. And, luciferase reporter assay to verify the relationship of miR-34a and the Cdc42-WASP-Arp2/3 pathway.

**Results:**

overexpression of miR-34a could induce ROS production and apoptosis, decrease the expression levels of DOCK8, p-WASP, WASP, Arp2, Arp3, and increase F-actin’s expression. Meanwhile, knockdown of miR-34a could decrease ROS production and apoptosis, increase the expression of DOCK8, p-WASP, WASP, Arp2, Arp3, and decrease F-actin’s expression. Immunofluorescence staining showed aberrant miR-34a and Cdc42-WASP-Arp2/3 pathway could induce F-actin membrane transfer. Luciferase reporter assay indicated that DOCK8 was a direct target gene of miR-34a.

**Conclusion:**

These data indicates miR-34a may induce neutrophil apoptosis by regulating Cdc42-WASP-Arp2/3 pathway-mediated F-actin remodeling and ROS production.

## 1. Introduction

Myelodysplastic syndrome (MDS) is a heterogeneous group of clonal disorders characterized by a decrease in neutrophil count, ineffective hematopoiesis, and high risk of conversion to leukemia [1, 2]. Most notably, the quantitative reduction and functional defects of neutrophils lead to reduced bactericidal and fungicidal activities, which may cause serious infections [3, 4]. Recently, clinical trials in MDS have proven that the application of hemopoietic growth factors can increase the neutrophil count and function [5]. However, the molecular basis of neutrophil quantitative reduction has yet to be clarified.

MicroRNAs (miRNAs) are negative regulators of the expression of genes involved in hematopoiesis. The roles of miRNAs in the pathogenesis of MDS and the transformation into acute myelocytic leukemia (AML) has been verified in many reports [6, 7], including miR-21, miR-194-5p and miR-29b [8–10]. miR-34a can inhibit proliferation by inducing apoptosis, meanwhile suppress the malignant cell lines’ migration and invasion [11]. Some damaged progenitors will die because of the proapoptotic feature of miR-34a, while some will differentiated into neutrophils [12]. In early MDS, the expression of miR-34a is abnormal and the upregulation of proapoptotic miR-34a contributes to the increased apoptosis of hematopoietic stem cells [13, 14]. Previous research by our team verified that miR-34a was markedly increased in MDS neutrophils, and ectopically introduced miR-34a significantly attenuated migration but enhanced degranulation [15]. In another report, we identified overexpression of c-Fos-targeting miR-34a as the cause of MDS-derived neutrophilic granulocyte impairment and showed that c-Fos reduction contributes to TNF-α overproduction via overexpression of miR-34a under inflammatory stimuli in MDS [16]. Therefore, miR-34a-induced apoptosis may be related to the reduction in neutrophil counts in MDS patients. However, the roles of miR-34a in the neutrophil count reduction in MDS are rarely studied, and the mechanism is still unclear.

In normal signaling, upstream cues load Cdc42 with the GTP nucleotide, which induces the protein to bind Wiskott Aldrich syndrome protein (WASP) with high affinity, and then the Cdc42(GTP)-WASP complex activates the actin nucleation factor Arp2/3 complex, thereby causing assembly of new actin filaments *in vitro* and *in vivo* [17, 18]. Researchers found that F-actin is closely related to cell apoptosis and the abnormal remodeling of F-actin can induce the accumulation of reactive oxygen species (ROS) in yeast cells, thereby inducing cell apoptosis [19]. In neutrophils of MDS patients, it have been verified that the remodeling of F-actin and the production of ROS are related [20].

Based on our previous study and other researchers’ reports, we hypothesized that miR-34a might regulate apoptosis via the Cdc42-WASP-Arp2/3 pathway and F-actin remodeling. The HL-60 cell line, derived from a patient with acute promyelocytic leukemia, consists predominantly (>90%) of promyelocytes, which ectopically overexpressed miR-34a and MDS granulocytes [15, 16, 21]. In this study, we introduced miR-34a mimics and its inhibitor, and performed Cdc42-WASP-Arp2/3 pathway inhibitor (ML141) and activator (CN02) treatments in neutrophil-like differentiated HL60 (dHL60) cells (which is a cell line used to study MDS) to ultimately clarify the molecular basis of aberrant miR-34a expression on neutrophil counts.

## 2. Materials and method

### 2.1. Cells and reagents

The human leukemic cell line HL60 was purchased from the Chinese Academy of Sciences Cell Bank (http://www.cellbank.org.cn/index.asp) and cultured at 37°C in RPMI 1640 supplemented with 10% heat-inactivated fetal bovine serum in a 5% CO_2_ atmosphere. Neutrophil-like dHL60 cells were induced to differentiate by culturing for 48 h in medium supplemented with 500 mM dibutyryl cAMP (dbcAMP) (Sigma-Aldrich, U.S.A.). The primary antibodies were obtained from Abcam (U.S.A.).

### 2.2. Cell transfection

miR-34a mimics/inhibitor and their negative sequences(NC) were prepared by Shanghai GenePharma Co., Ltd. (Table 1). 2 × 10^6^ cells/well were transfected with the above sequences using Lipofectamine 2000 (Invitrogen, U.S.A.). Finally, qRT-PCR was employed to evaluate the efficiency of transfection.

**Table 1.** Primer sequences in qRT-PCR and cell transfection.

Names	Sequence (5 ‘-3’)
miR-34a reverse transcription primer	GTCGTATCCAGTGCGTGTCGTGGAGTCGGCAATTGCACTGGATACGACACAACCAG
Universal reverse primer	CAGTGCGTGTCGTGGAGT
miR-34a	CGGTGGCAGTGTCTTAGCT
U6-F	CTCGCTTCGGCAGCACA
U6-R	AACGCTTCACGAATTTGCGT
miR-34a mimics	UGGCAGUGUCUUAGCUGGUUGU
miR-34a inhibitor	ACAACCAGCUAAGACACUGCCA
NC	GGGAGUGAAGACACGGAGCCAGA

Note: F means forward primer, R means reverse primer.

### 2.3. Quantitative real-time PCR (qRT-PCR)

Total RNA was extracted from cells and then was synthesized the cDNA. qRT-PCR was performed using a 7500 real-time PCR system (Applied Biosystems). The qRT-PCR cycling program setting was: pre-denaturation at 95°C for 5 min, followed by 39 cycles at 95°C for 10 s and 60°C for 34 s. The primers of the qRT-PCR are listed in Table 1. The mRNA levels were normalized to GAPDH/U6 expression levels and calculated using the 2^-ΔΔCq^ formula.

### 2.4. ROS measurement

The levels of ROS was measured by chloromethyl-2’,7’ dichlorodihydrofluorescein diacetate (CM-H2DCFDA) staining. Briefly, 2×10^5^ cells were collected, centrifuged, suspended and then stained with 10 µM CM-H2DCFDA for 50 min. After washing two times, FACSCalibur flow cytometer (BD, Accuri C6, U.S.A.) measured the fluorescence at 538 nm. The percentage of DCF-positive cells and their mean fluorescence intensity (MFI) were statistically analyzed.

### 2.5. SOD, CAT and GSH-Px measurement

Superoxide dismutase (SOD), catalase (CAT) and glutathione peroxidase (GSH-PX) measurement were performed using biochemical detection kits. Cell sample preparation and detection steps were operated according to the manufacturer's instructions. Repeat three times per test for each group. The kits came from Nanjing Jiancheng Bioengineering Institute (Nanjing, China).

### 2.6. Apoptosis assay

After successful transfection for 48 h, we collected the transfected cells and determined their apoptosis using an apoptosis detection kit (BD, #556547, U.S.A.). Briefly, the cells were washed two times with PBS and then resuspended in Annexin V binding buffer at a density of 1×10^5^ cells/mL. Cells were simultaneously stained with fluorescein isothiocyanate (FITC)-labeled Annexin V and propidium iodide (PI) for 15 min in the dark. Then, a FACSCalibur flow cytometer (BD, Accuri C6, U.S.A.) was employed to detect and analyze the percentage of Annexin V+ and PI+ cells. The sum of Annexin V+ percentage and PI+ percentage is the apoptosis rate.

### 2.7. Cdc42 activator and inhibitor treatment

Cdc42 activator (Cytoskeleton, #CN02, U.S.A.) is useful for efficient activation of Cdc42. The Cdc42 inhibitor ML141 (Sigma, #217708, U.S.A.) is a highly potent and selective inhibitor of Cdc42. More than 2×10^6^ cells were cultured with 0.1 units/mL CN02 for 5 min. Then, the cells were exchanged into CN02-free medium and cultured with 0.3 mM H_2_O_2_ for 4 h. Alternatively, more than 2×10^6^ cells were cultured with 2.5 µM ML141 for 48 h. Then, exchanged the cells into ML141-free medium and cultured with 0.3 mM H_2_O_2_ for 4 h. Finally, measured the expression levels of apoptosis, ROS levels and related proteins.

### 2.8. Western blotting

After lysis and quantification of total protein concentration, 50 μg total protein was added to the wells of sodium dodecyl sulfate-polyacrylamide gel electrophoresis gels, and incubated with the primary antibodies at 37°C for 2 h. The primary antibodies were the anti-DOCK8 (1:10000, ab175208), anti-WASP (1:500, ab180816), anti-p-WASP (1:500, ab59278), anti-Arp2 (1:500, ab128934), anti-Arp3 (1:500, ab151729), and anti-F-actin (1:500, ab205) antibodies. Finally, a ChemiDoc image analysis system (Bio-Rad Laboratories, Inc.) was used to analyze and quantify the relative protein levels. And the levels of GAPDH protein were used for normalization.

### 2.9. Immunofluorescence analysis

After fixing and permeating with 4% paraformaldehyde 0.5% Triton X-100 respectively, 1×10^6^ cells samples were incubated with anti-F-actin primary antibodies (1:100, ab205) for 2 h. Then, incubated the cells with Alexa Fluor 488-conjugated IgG (ZSGB-BIO, ZF-0512, China) at 37°C for 1 h. DAPI (10 μg/ml) was used for cellular nuclei staining, and then took photos at 400× magnification. Image-Pro Plus 6.0 software (Media Cybernetics, U.S.A.) to calculate the mean optical density (MOD) of F-actin and the ratio of membrane and cytosolic F-actin expression.

### 2.10. Luciferase reporter assay

The binding sites between miR-34a and dedicator of cytokinesis 8 (DOCK8) (https://cm.jefferson.edu/rna22/Interactive/RNA22 Controller) were predicted using software RNA Central and RNA22 v2. The full-length 3’UTR of human DOCK8 mRNA was cloned into the pGL3 promoter vector to construct the luciferase reporter plasmid. The DOCK8 3’UTR was mutated by site-directed mutagenesis. For the transfection, 2×10^6^ cells were transfected using Lipofectamine 2000 (Invitrogen) with 150 nM miR-34a mimics or inhibitor. After 24 h, cells were co-transfected with 100 ng luciferase reporter plasmid. Prepared cell extracts after 48 h later, and then detected the fluorescence intensity and calculated the relative luciferase activity.

### 2.11. Statistical methods

Data are listed as the mean ± standard deviation (SD). Figures were graphed using GraphPad Prism 5 (GraphPad Software, U.S.A.). Statistically significant differences between groups were determined by one-way analysis of variance (ANOVA) with the Bonferroni post hoc test. *P *< 0.05 were considered statistically significant.

## 3. Results

### 3.1. miR-34a regulates ROS production, apoptosis and Cdc42-WASP-Arp2/3 pathway in neutrophil-like dHL60 cells

**Figure 1.**
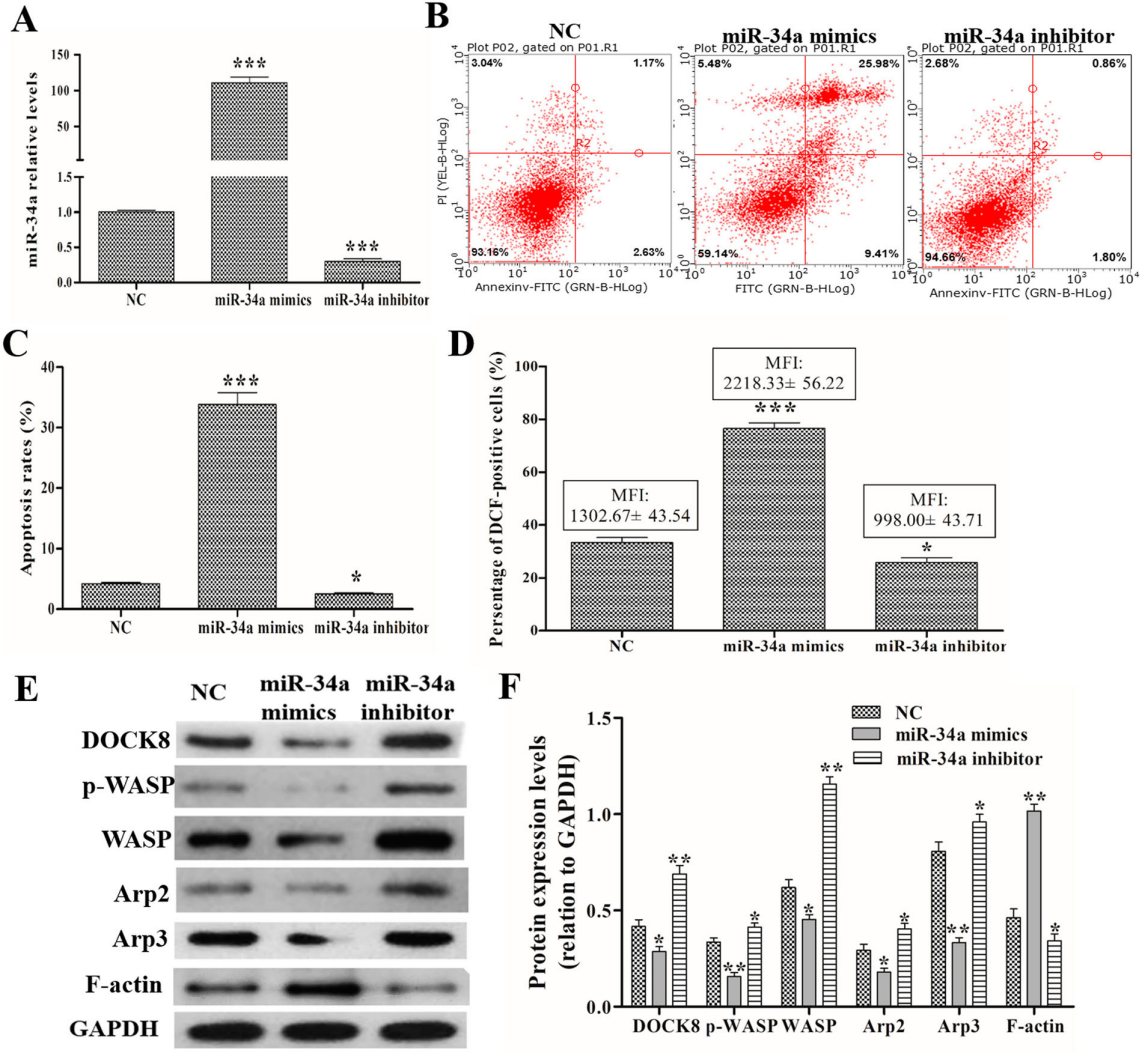
Effects of miR-34a on ROS production, apoptosis and related protein expression. Cells were preformed the miR-34a mimics/inhibitor transfection. (A) qRT-PCR to detect the expression levels of miR-34a. (B and C) FACSCalibur flow cytometer images and statistical analysis of apoptosis rate. (D) Statistical analysis of the percentage of DCF-positive cells and their MFI. (E and F) Western blot analysis of the expression levels of DOCK8, p-WASP, WASP, Arp2 and Arp3. Compared with the NC group, *P < 0.05, **P < 0.01, ***P < 0.001.

After miR-34a mimics/inhibitor transfection, qRT-PCR detected the expression levels of miR-34a and found that miR-34a mimics transfection could significantly increase miR-34a levels and miR-34a inhibitor transfection could significantly decrease miR-34a levels compared with those in the negative transfection cells (NC group) (Figure 1A). After efficient transfection, a FACSCalibur flow cytometer was used to measure ROS levels and apoptosis rate. As shown in Figure 1(B and C), compared with the NC group, the overexpression of miR-34a could increase the percentage of apoptosis cells, while knockdown of miR-34a could reduce the percentage of apoptosis cells. As shown in Figure 1(D), compared with the NC group, the overexpression of miR-34a could increase the percentage of DCF-positive cells and their MFI, but knockdown of miR-34a could reduce the percentage of DCF-positive cells and their MFI. Additionally, Western blotting found that overexpression of miR-34a decreased DOCK8, p-WASP, WASP, Arp2, Arp3 expression, and knockdown of miR-34a increased DOCK8, p-WASP, WASP, Arp2, Arp3 expression compared with that in the NC group (Figure 1E and F). Furthermore, the overexpression of miR-34a could decrease the levels of SOD, CAT and GSH-PX, and knockdown of miR-34a could increase their levels (shown at Table 2). These data indicate that miR-34a regulates ROS production, apoptosis and Cdc42-WASP-Arp2/3 pathway in neutrophil-like dHL60 cells.

**Table 2.** Levels of SOD, CAT and GSH-PX after miR-34a mimics/inhibitor transfection.

	NC	miR-34a mimics	miR-34a inhibitor
SOD (IU/ml)	51.50 ± 1.28	25.53 ± 0.93***	75.93 ± 0.83***
CAT (IU/mgprot)	11.00 ± 0.53	5.10 ± 0.53***	15.20 ± 0.80***
GSH-PX (IU)	78.23 ± 1.35	63.50 ± 0.95***	90.10 ± 1.35***

Compared with the NC group, ***P < 0.001.

### 3.2. Cdc42-WASP-Arp2/3 pathway regulates ROS production and apoptosis in neutrophil-like dHL60 cells

**Figure 2.**
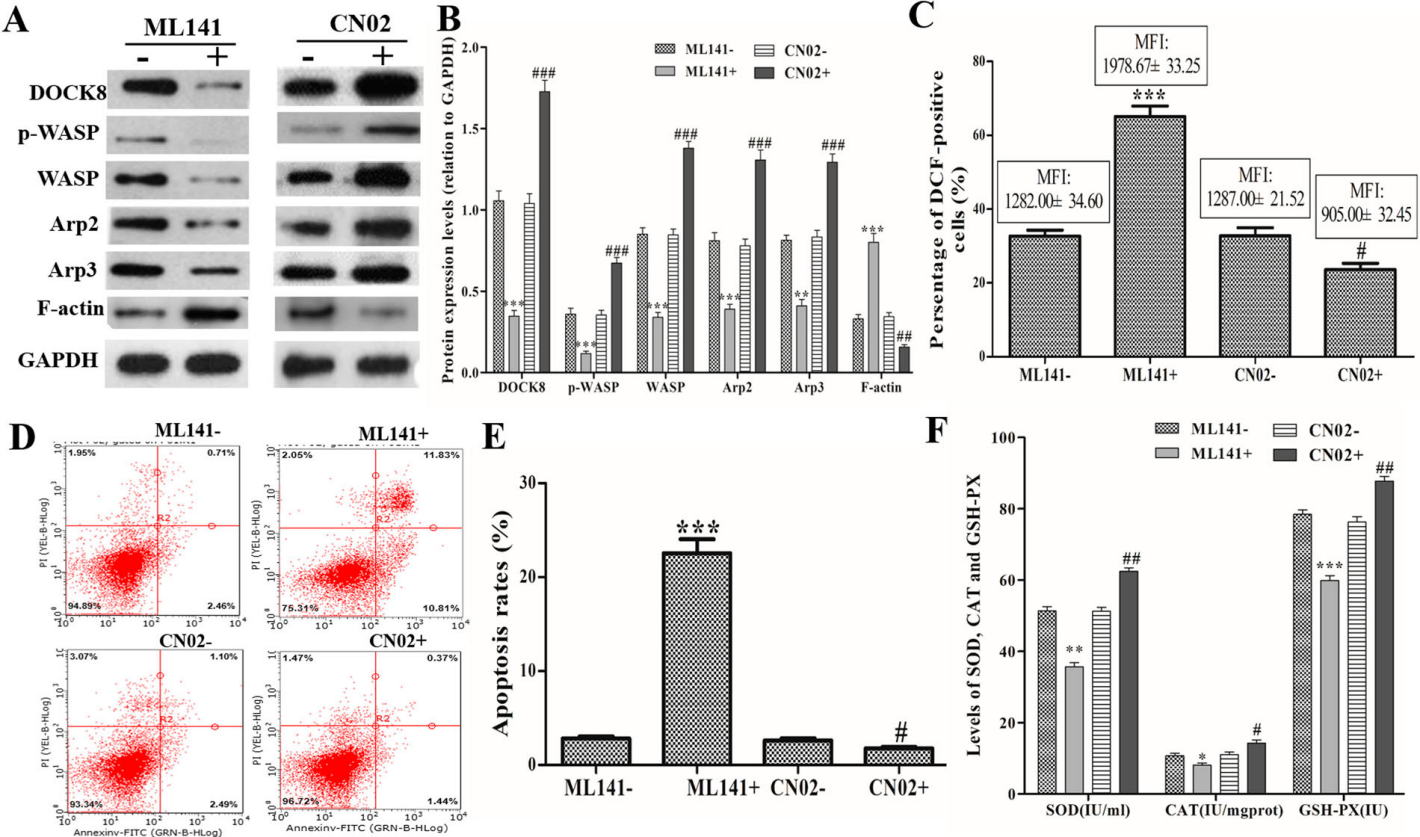
Effects of the Cdc42-WASP-Arp2/3 pathway on ROS production and apoptosis. Cells were treated with Cdc42-WASP-Arp2/3 pathway inhibitor (ML141) and activator (CN02) treatment. (A and B) Western blot to detect the expression levels of DOCK8, p-WASP, WASP, Arp2, Arp3 and F-actin. (C) Statistical analysis of the percentage of DCF-positive cells and their MFI. (D and E) FACSCalibur flow cytometer images and statistical analysis of apoptosis rate. (F) Levels of SOD, CAT and GSH-PX in each group. Compared with the ML141-untreated (ML141-) group, *P < 0.05, **P < 0.01, ***P < 0.001. Compared with the CN02-untreated (CN02-) group, ^#^*P *< 0.05, ^##^*P *< 0.01, ^###^*P *< 0.001.

After Cdc42-WASP-Arp2/3 pathway inhibitor (ML141) and activator (CN02) treatment, Western blot showed that ML141 treatment could decrease DOCK8, p-WASP, WASP, Arp2, Arp3 expression, and CN02 treatment could increase DOCK8, p-WASP, WASP, Arp2, Arp3 expression compared with those in the untreated cells (Figure 2A and B). In addition, Cdc42 activator treatment (ML141 + group) could increase the percentage of DCF-positive cells and their MFI, while Cdc42 inhibitor treatment (CN02 + group) could reduce the percentage of DCF-positive cells and their MFI (Figure 2C). Apoptosis assay showed that ML141 treatment could increase the apoptosis, and CN02 treatment could reduce the apoptosis (Figure 2D and E). Furthermore, ML141 treatment could decrease the levels of SOD, CAT and GSH-PX, and CN02 treatment could increase their levels (Figure 2F). These data indicated that the Cdc42-WASP-Arp2/3 pathway regulates ROS production and apoptosis in neutrophil-like dHL60 cells.

### 3.3. miR-34a regulates F-actin expression/remodeling in neutrophil-like dHL60 cells

**Figure 3.**
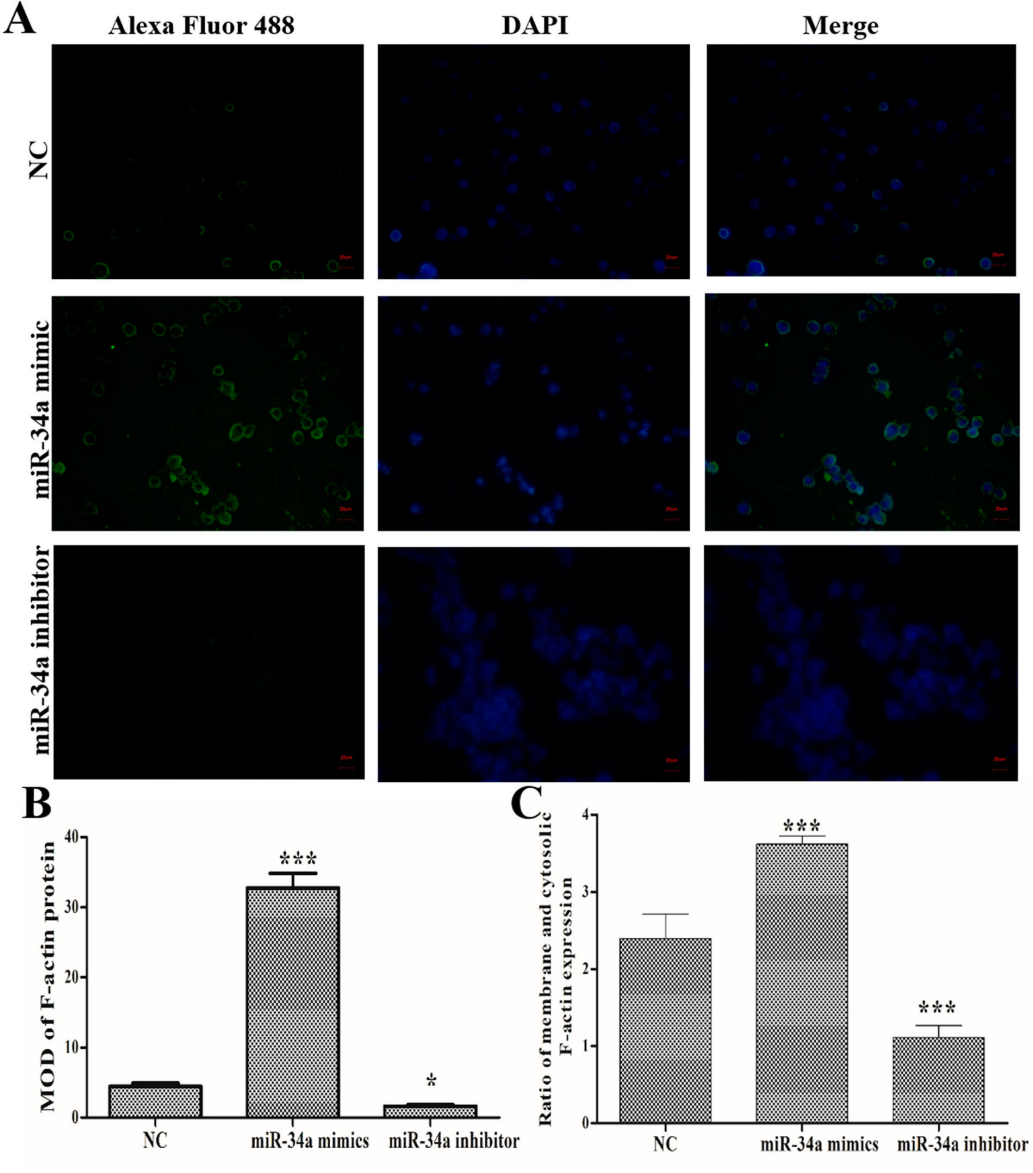
Effects of miR-34a on F-actin expression/remodeling. (A) Immunofluorescence staining to determine the expression and distribution of F-actin (Magnification: 400×). (B) MOD of F-actin protein expression. (C) Ratio of membrane and cytosolic F-actin expression. Compared with the NC group, *P < 0.05, ***P < 0.001.

Using immunofluorescence staining (Figure 3A), we found that miR-34a mimics transfection induced an increase in F-actin expression and miR-34a inhibitor transfection induced a decrease in F-actin expression compared with NC group (Figure 3B). Importantly, while miR-34a mimics’ transfection increased the expression of F-actin protein, it also promoted F-actin transfer to the periphery of the cell. Compared with NC group, miR-34a inhibitor transfection inhibited F-actin transfer to the periphery of the cells (Figure 3C). These data indicated that miR-34a could regulate F-actin expression and remodeling.

### 3.4. Cdc42-WASP-Arp2/3 pathway regulates F-actin expression/remodeling in neutrophil-like dHL60 cells

**Figure 4.**
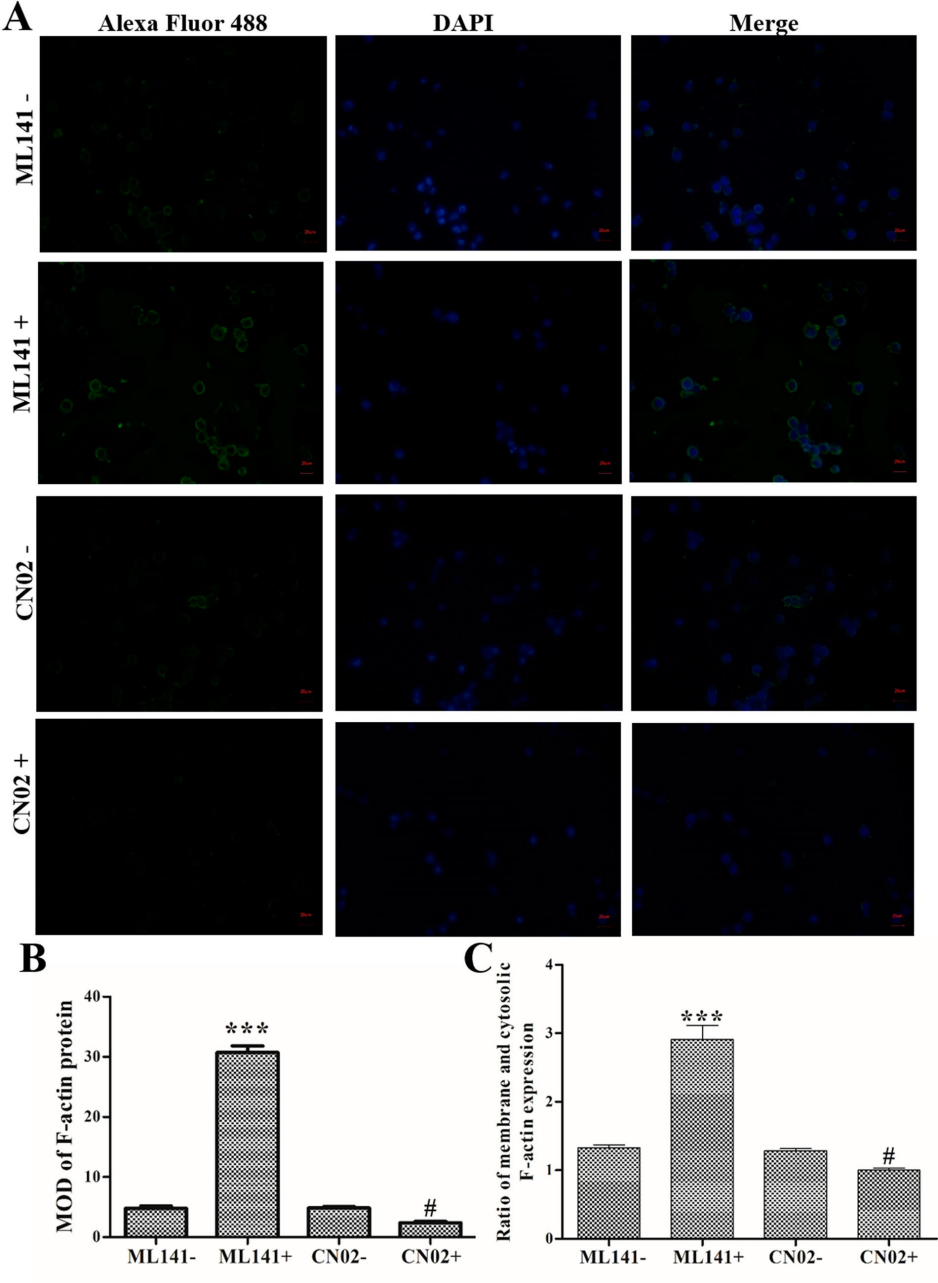
Effects of Cdc42-WASP-Arp2/3 pathway on F-actin expression/remodeling. Cells were treated with ML141 and CN02 respectively. (A) Immunofluorescence staining to determine the expression and distribution of F-actin (Magnification: 400×). (B) MOD of F-actin protein. (C) Ratio of membrane and cytosolic F-actin expression. Compared with ML141- group, ***P < 0.001. Compared with the CN02- group, ^#^*P *< 0.05.

Meanwhile, immunofluorescence staining found that ML141 treatment induced an increase in F-actin expression, and CN02 treatment induced a decrease in F-actin expression compared with ML141- and CN02- group, respectively (*P *< 0.05, Figure 4B). Additionally, ML141 treatment promoted F-actin transfer to the periphery of the cell, and CN02 treatment inhibited F-actin transfer to the periphery of the cells (Figure 4C). These data indicated that Cdc42-WASP-Arp2/3 pathway could regulate F-actin expression and remodeling.

### 3.5. miR-34a induces apoptosis by regulating Cdc42-WASP-Arp2/3 pathway-mediated F-actin remodeling in neutrophil-like dHL60 cells

**Figure 5.**
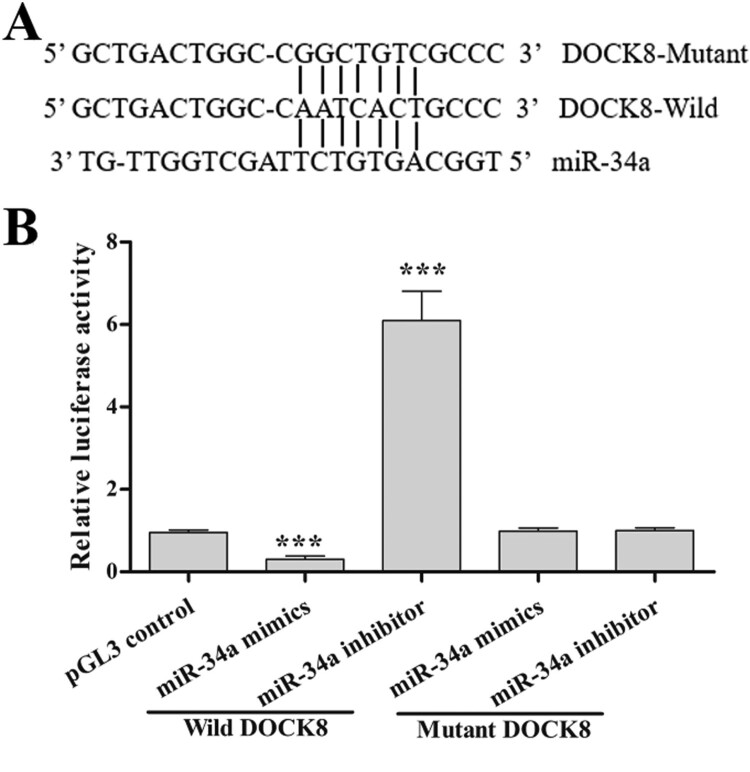
DOCK8 is a direct target gene of miR-34a. (A) The predicted binding sites between miR-34a and DOCK8. (B) The relative luciferase activity in each group. Compared with the control group, ***P < 0.001.

To verify the relationship of miR-34a and the Cdc42-WASP-Arp2/3 pathway, we characterized the binding site of miR-34a in the 3’UTR of DOCK8 mRNA (Figure 5A). Luciferase reporter assays (Figure 5B) indicated that miR-34a overexpression (miR-34a mimics’ transfection) decreased DOCK8 transcriptional activity (decreased relative luciferase activity) and miR-34a knockdown (miR-34a inhibitor transfection) increased DOCK8 transcriptional activity (increased relative luciferase activity). Furthermore, compared with the control group, the mutant reporter co-transfected with mutant DOCK8 did not show a significant increase/decrease in the relative luciferase activity. These data suggest that DOCK8 is a direct target gene of miR-34a. Taken together, miR-34a regulates apoptosis through regulation of F-actin remodeling via the Cdc42-WASP-Arp2/3 pathway.

## 4. Discussion

In early MDS, the miR-34a expression is abnormal and its upregulation increased the hematopoietic stem cell apoptosis and neutrophil migration [13–16]. The introduction of miR-34a decreased the active form of Cdc42 and DOCK8. DOCK8 is involved in the migration of miR-34a-mediated neutrophil [15]. A decrease in DOCK8, p-WASP, WASP, Arp2 and Arp3 levels by the miR-34a overexpression transfection and an increase in DOCK8, p-WASP, WASP, Arp2 and Arp3 levels by the miR-34a knockdown transfection indicated that miR-34a might target the Cdc42-WASP-Arp2/3 pathway. Our luciferase reporter assay verified that miR-34a targeted DOCK8, and thus targeted and regulated the Cdc42-WASP-Arp2/3 pathway in neutrophils. Is the Cdc42-WASP-Arp2/3 pathway involved in miR-34a-regulated apoptosis of neutrophils? Inactivation (or inhibition) of the Cdc42-WASP-Arp2/3 pathway could induce ROS production and apoptosis, and activation of the Cdc42-WASP-Arp2/3 pathway could reduce ROS production and apoptosis. Therefore, we demonstrated that miR-34a induces neutrophil apoptosis via the Cdc42-WASP-Arp2/3 pathway.

Neutrophil effector functions are dependent on the organization of the actin cytoskeleton [22]. There are 2 forms of actin, a filamentous form (F-actin) and a monomeric form (G-actin), and rapid conversion of G-actin to F-actin occurs under the stimulation of neutrophils with chemotactic factors [20]. The dynamics of the actin cytoskeleton lead to a loss of mitochondrial membrane potential, resulting in ROS production and apoptosis in budding yeast, and the release of ROS by mitochondria instigates the pathways of programmed cell death in eukaryotic cells [23]. *In vitro* experiment, the actin disruption agent latrunculin B (LB) induced apoptosis by upregulating COX-2 and NF-kB activation and producing ROS [24]. Rho family have been implicated as important signaling intermediates that link cell surface signals to the actin cytoskeleton, and the actin cytoskeleton links the Rho family GTPase Cdc42 to the actin-nucleating Arp2/3 complex through N-WASP [25]. Immunofluorescence staining found that aberrant miR-34a expression and activation/inhibition of the Cdc42-WASP-Arp2/3 pathway regulate F-actin expression and distribution, indicating that F-actin remodeling is regulated by miR-34a and Cdc42-WASP-Arp2/3 pathway. Correspondingly, the apoptosis increased with the remodeling of the F-actin protein.

Under both physiologic and pathologic conditions, ROS and mitochondria plays a pivotal role in apoptosis induction [26]. Excess ROS can cause serious damage to many biological macromolecules, whose oxidation leads to their biological properties damage and eventually to cell death [27]. Researchers also have found that ROS are mostly generated by the impairment of the mitochondrial respiratory chain, ROS generation is accompanied by cytochrome c release, caspase-8 activation, etc, which would trigger apoptosis [28]. In human lymphocytes, hypoxia/reoxygenation (H/R) induced apoptosis through ROS production and mitochondrial membrane potential collapse [29]. Pretreatment with 1 mM N-acetylcysteine (NAC), a well-known ROS scavenger, can attenuate apoptosis induced by the pathologic conditions [30]. In this study, the high levels of ROS caused by miR-34a mimics transfection was accompanied by a high apoptosis rate, meanwhile the low levels of ROS caused by miR-34a inhibitor transfection was accompanied by a low apoptosis rate. And, Changes in ROS levels due to Cdc42-WASP-Arp2/3 pathway activation/inactivation were also proportional to the apoptosis rate. These data indicate that miR-34a and its regulated Cdc42-WASP-Arp2/3 pathway are involved in ROS-mediated apoptosis. Based on the promoting effect of high levels of ROS on apoptosis, the use of ROS scavengers may provide research ideas for the reduction of neutrophil counts caused by abnormally high expression of miR-34a.

## 5. Conclusions

**Figure 6.**
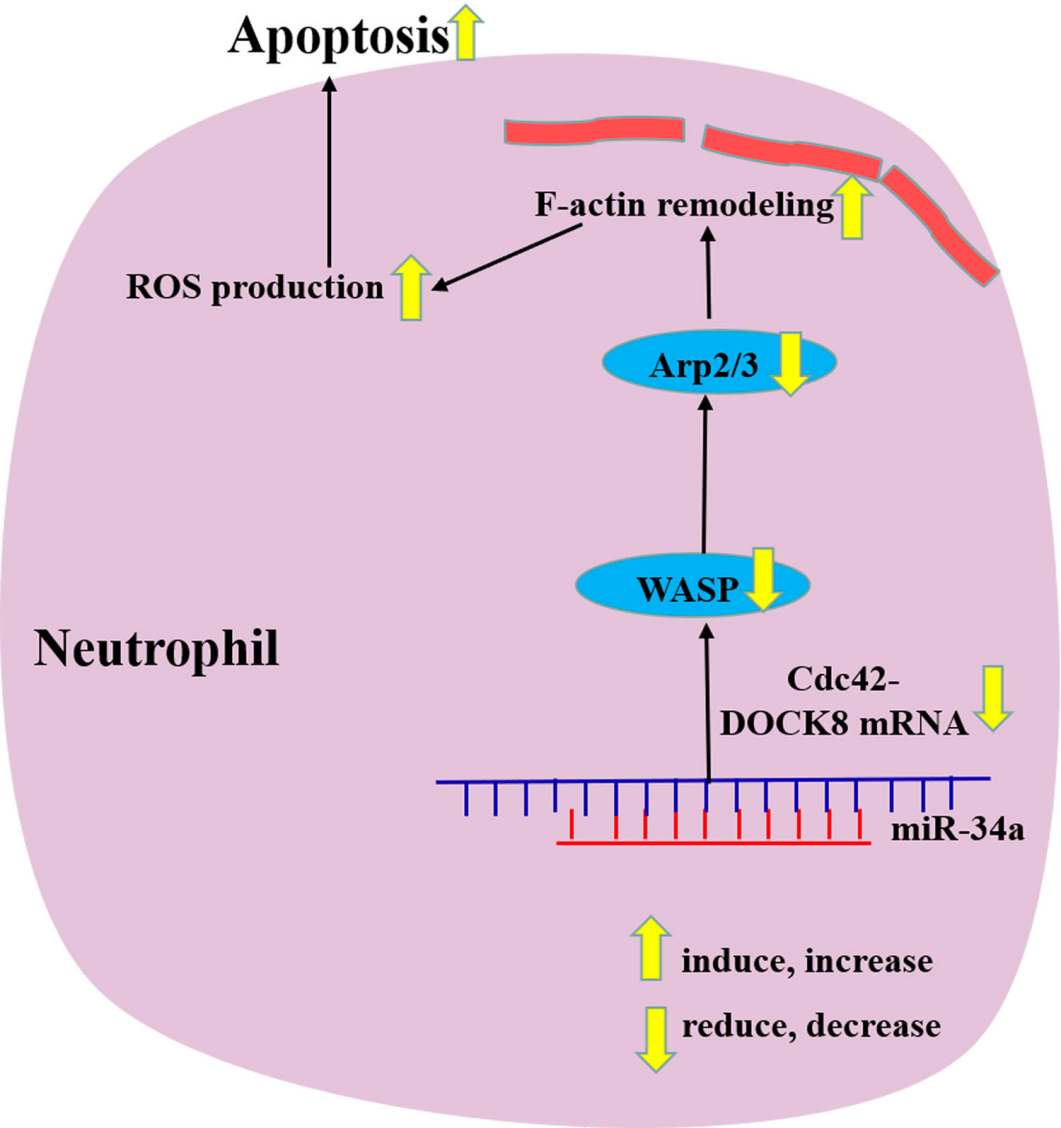
Schematic diagram of miR-34a regulating neutrophil apoptosis in MDS.

In this study, we identified the apoptosis mechanisms of neutrophils by aberrantly increasing/decreasing miR-34a and demonstrated that miR-34a induces neutrophil-like dHL60 cell apoptosis, which may be related to Cdc42-WASP-Arp2/3 pathway-mediated F-actin remodeling and ROS production (Figure 6). These findings provide new insights into the pathophysiology behind the quantitative reduction in MDS neutrophils. In the future study, we will try to use different cells (not only HL60) or study different miRNAs and their pathways to reveal the more mechanism of neutrophils apoptosis in MDS.

## Data Availability

The datasets used and/or analyzed during the current study appear in the submitted article.
